# The Molecular Theory of Liquid Nanodroplets Energetics in Aerosols

**DOI:** 10.3390/e23010013

**Published:** 2020-12-24

**Authors:** Sergii D. Kaim

**Affiliations:** Faculty of Electrical Engineering Automatic Control and Informatics, Opole University of Technology, ul. Prószkowska 76, 45-758 Opole, Poland; s.kaim@po.edu.pl; Tel.: +48-77-537-809-228

**Keywords:** nanodroplet aerosols, the effective Hamiltonian, surface energy, atom–nanodroplet interaction energy, interaction energy of two nanodroplets, size dependence, adhesion energy of a molecular complex and a liquid nanodroplet

## Abstract

Studies of the coronavirus SARS-CoV-2 spread mechanisms indicate that the main mechanism is associated with the spread in the atmosphere of micro- and nanodroplets of liquid with an active agent. However, the molecular theory of aerosols of microdroplets in gases remains poorly developed. In this work, the energy properties of aerosol nanodroplets of simple liquids suspended in a gas were studied within the framework of molecular theory. The three components of the effective aerosol Hamiltonian were investigated: (1) the interaction energy of an individual atom with a liquid nanodroplet; (2) the surface energy of liquid nanodroplet; and (3) the interaction energy of two liquid nanodroplets. The size dependence of all contributions was investigated. The pairwise interparticle interactions and pairwise interparticle correlations were accounted for to study the nanodroplet properties using the Fowler approximation. In this paper, the problem of the adhesion energy calculation of a molecular complex and a liquid nanodroplet is discussed. The derived effective Hamiltonian is generic and can be used for the cases of multicomponent nano-aerosols and to account for particle size distributions.

## 1. Introduction

The rapid spread of coronavirus SARS-CoV-2 has become an investigation subject for numerous scientists. The existing data exposed the ability of a virus to be transmitted in an airborne manner as dispersed droplets that contain the infective agent [1,2,3,4,5,6]. Airborne transmission is defined by the World Health Organization (WHO) as the spread of invective agents through suspended droplets in the air, which stay infective for long periods of time and may travel long distances [7].

An important physical aspect in the problem of virus spread is the interaction of nanoparticles (virions) and nanodroplets with molecular structures of different media. For these types of problems, an important characteristic is the size dependence of energetic properties of nanodroplets and nanoparticles at interactions with different media structures. The calculations of the adhesion energy of nanoparticles to the different structures, in addition to the calculations of energetic characteristics for aerosol nanodroplets, require an application of statistical physics methods. An investigation of equilibrium and nonequilibrium properties of droplets and aerosols with liquid nanodroplets can be performed within the framework of classical statistical mechanics. The nanodroplets may reveal implicit collective properties and self-organization into structures at a macroscopic level [8]. The behavior of an isolated nanodroplet can be simulated by means of molecular dynamics [9]. 

The typical complications in the theoretical analysis of nanosystems are conditioned by the necessity to account for the surface terms for all of its equilibrium and nonequilibrium properties. At nanometer scales, an abrupt change of all system characteristics takes place near the surface. The intermolecular forces act at similar scales.

The contemporary state of theoretical studies of the structure and properties of liquid nanodroplets within the framework of molecular–kinetic theory has been summarized in the works [10,11,12]. A statistical approach to the study of volumetric properties of equilibrium and nonequilibrium homogeneous systems shows the importance of accounting for the paired interparticle interactions and correlations, which contain determinative terms in all properties [13,14,15,16]. For inhomogeneous liquids in statistical theory, it is important to take into account the one-particle distribution functions and the effective one-particle potentials, which are determined by paired interparticle interactions and correlations. The contribution of the one-particle effects to the properties of inhomogeneous systems corresponds to the accounting of surface terms.

The current study, using the correlation theory of inhomogeneous liquids, investigated the equilibrium properties of nanodroplets as components of aerosol systems. Thus, there was a need to use approximations that allowed a reasonable comparison of theoretical and experimental results to be made. The properties of aerosol systems were determined by their interacting components, i.e., nanodroplets and gas. There exist many phenomena at the molecular level that lack explanation. Under these conditions, it is difficult to predict the behavior of nano-objects and their assemblies, or to control these nanosystems. For the investigated aerosol systems, a fundamental role is played by the energy characteristics of the separate nanodroplets, their interaction energies with the surrounding gas, and the paired interactions of nanodroplets. Investigation of the pointed energy characteristics is a major focus of the current article.

## 2. The Calculation of the Molecule Interaction Energy with Liquid Nanodroplet

Formulation and resolution of the problems related to the interaction of isolated atoms and molecules with the heterogeneity of the condensed system (surface, new phase origins, phase transition fronts) are essential in constructing a microscopic theory of first-order phase transitions, in addition to a microscopic theory of equilibrium and kinetic properties of the surface and interphase boundaries. A review of the current state of the problems of atom interactions with an inhomogeneous environment can be found in [10,11,12,17]. However, the problems of size dependencies of the interaction energy of atoms and nanosized condensed systems, with accounting for interatomic correlations, remain unsolved.

In statistical mechanics of condensed matter, as a rule, the potential interaction energy of two atoms or two molecules is assumed to be known. An approximation of pair additive potentials is widely used to describe the energies of interatomic interactions [10,11,12,13,14,15,16]. Among the most used potentials of interatomic interaction, Lennard–Jones potential and its generalizations, Morse potential, hard-sphere potential, and soft-sphere potential should be noted [10,11,12,13,14,15,16].

Calculation of the interaction energy of macroscopic bodies with different geometries in continual approximation, including accounting for van der Waals forces, is described in [11,12]. The calculations of atom interaction energy with an object outside the framework of continual approximation, when it is necessary to account for the repulsion of atoms and pair correlation in its position, have attracted significant interest. For an interaction of isolated atoms with solid objects, the continual approximation is correct and accounts for the repulsion of an atom from the solid body atoms (without accounting for an atomistic structure of the solid body and the possibility of atoms penetrating into the body). However, in the case of atom–liquid interaction, this approach is insufficient. In the equilibrium system of liquid–vapor, an interchange of atoms between both phases takes place, and the equilibrium is dynamic. To take account of an interchange between two phases, it is necessary to equally account for a discrete structure of both the vapor and liquid. A mathematical technique to describe this process should be similar for both phases.

A wide overview of the literature regarding the interaction of atoms and macroscopic bodies [11] points to a range of unsolved problems, which are essential for understanding the processes in the nanoscale systems. A topical problem is the calculation of the interaction energy of atoms with droplets and the solid bodies of nanoscale size, including accounting for the surface curvature and repulsive effects, and corresponding correlations in their positions.

The goal of the current section is to present the model calculations of the interaction energy between the atoms and nanodroplets of a simple liquid. The foundation of these calculations is based on the expression for the energy of an inhomogeneous liquid within the framework of the distribution function method of groups of particles, which takes into account all paired interatomic interactions and correlations. The paired interatomic interactions were described using the Lennard–Jones potential. The structure of the droplets was modeled using a Fowler approximation (the step profile of atoms’ density in the droplets) [18,19]. The calculations are performed for the cases in which the atom is located outside, inside, and on the surface of the droplet. For the geometry dispositions of the atom and droplet, which require accounting for the paired interatomic correlations, pair distribution functions within the framework of thermodynamic perturbation theory were used.

The potential energy of an inhomogeneous system of a pair of interacting particles, located in a volume V, can be written as [10,19]:

(1) $$E=\frac{1}{2}n_{0}^{2}\int_{V}d^{3}r_{1}\int_{V}d^{3}r_{2}F_{2}\left(r_{1},r_{2}\right)\Phi \left(r_{1},r_{2}\right)$$

where $\Phi \left(r_{1},r_{2}\right)$ is an interaction energy of the pair of atoms; is a pair distribution function of atoms inside an inhomogeneous liquid in a canonical Gibbs ensemble; and $n_{0}$ is the density of the number of particles. Formula (1) takes into account all paired interparticle interactions and correlations for the energy of the inhomogeneous liquid. The interaction energy of the volume element dV of the liquid with the remainder of the liquid can be written as:

(2) $$E_{el-liq}=n_{0}^{2}dV_{1}\int_{V}d^{3}r_{2}F_{2}\left(r_{1},r_{2}\right)\Phi \left(r_{1},r_{2}\right)$$

By choosing the volume element from the condition $dV_{1}n_{0}=1$, we obtain an expression for the interaction energy of an isolated atom with an inhomogeneous liquid. For the model calculations, a central symmetry potential of paired interaction of the atoms was used, and the following approximation for the pair distribution function inside the droplet:

(3) $$F_{2}\left(r_{1},r_{2}\right)≅F_{2}^{\left(0\right)}\left(\left|r_{1}-r_{2}\right|\right)\Theta \left(a-r_{1}\right)\Theta \left(a-r_{2}\right),$$

where $F_{2}^{\left(0\right)}\left(\left|r_{1}-r_{2}\right|\right)$ is a pair distribution function of atoms in homogeneous liquid; Θ(x) is the Heaviside step-function; a is the radius of the droplet.

**Figure 1:**
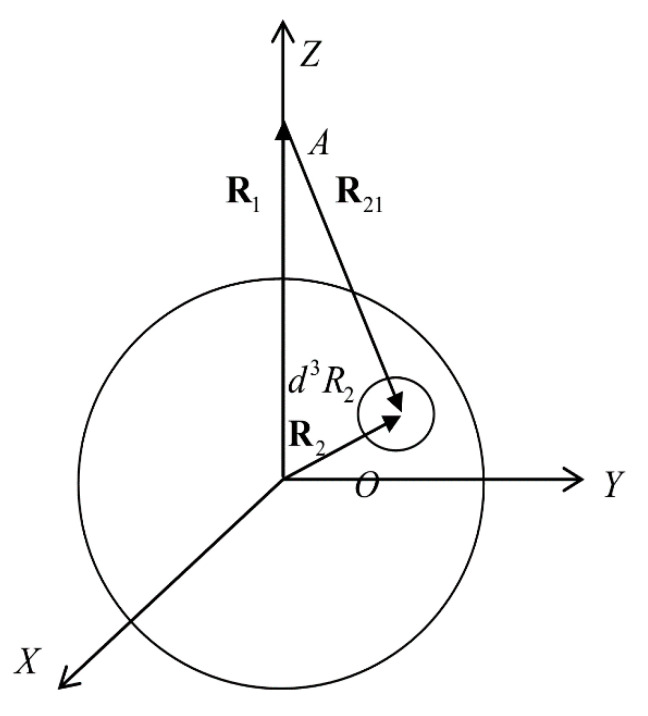
Geometry of atom and droplet location.

Figure 1 shows a droplet and atom at the point A. The radius vector $R_{1}$ indicates the location of the atom, interacting with the droplet, and radius vector $R_{2}$ indicates the location of the volume element. By introducing new integration variables via relation $R_{2}-R_{1}\equiv R_{12}$ and using spherical coordinates for integration (Figure 1), we obtain the following expression for the atom–droplet interaction:

(4) $$\begin{array}{c} E_{a-d}\left(R_{1},a\right)=n_{0}\int_{0}^{\infty }dRR^{2}\int_{0}^{\pi }\sin\Theta d\Theta \int_{0}^{2\pi }d\varphi \Phi \left(R\right)F_{2}^{\left(0\right)}\left(R\right)\times \\ \times \Theta \left(a-{\left(R_{1}^{2}+R^{2}+2R_{1}R\cos\Theta \right)}^{1/2}\right). \end{array}$$

After integration over spherical variables, the expression for the atom–droplet interaction energy takes the form:

(5) $$\begin{array}{c} E_{a-d}\left(R_{1},a\right)=\frac{\pi n_{0}}{R_{1}}\Theta \left(R_{1}-a\right)\int_{R-a}^{R+a}dRR\Phi \left(R\right)F_{2}^{\left(0\right)}\left(R\right)\times \\ \times \left[a^{2}-R_{1}^{2}-R^{2}+2RR_{1}\right] \end{array}$$

where $R_{1}$ is the distance from the atom to the center of the droplet. The obtained expression is valid for the distances $R_{1}\geq a$. From (5), we can see that the atom–droplet interaction potential is dependent on the geometrical size of the droplet and the thermodynamic parameters, such as density of the number of atoms and temperature. Formally, $E_{a-d}\left(R_{1},a\right)$ is a function of the potential of the paired interparticle interaction and pair distribution function of the atoms. The derived Expression (5) takes into account the kinematic conditions of the atoms’ arrangement outside the droplet.

If the atom is located at the surface of the droplet, then $R_{1}=a$ and the interaction energy expression takes the form:

(6) $$E_{a-d}\left(a,a\right)=\frac{\pi n_{0}}{a}\int_{0}^{2a}dRRF_{2}^{\left(0\right)}\left(R\right)\Phi \left(R\right)\left(2aR-R^{2}\right).$$

In this case, from (6), it is clearly seen that the atom–droplet interaction energy takes finite values, unlike in the case of continual approximations, in which no body structure is taken into account and density is assumed to be constant [11,12]. In the continual approximations, the atom energy at the surface of the droplet tends to infinity due to the repulsive forces acting on the atom from the droplet.

For the case in which the atom is located inside the droplet, similar calculations allow the interaction energy of the atom and droplet to be obtained:

(7) $$\begin{array}{c} E_{a-d}\left(R_{1},a\right)=4\pi n_{0}\Theta \left(a-R_{1}\right)\int_{0}^{a-R_{1}}dRR^{2}\Phi \left(R\right)F_{2}^{\left(0\right)}\left(R\right)+\\ +\frac{\pi n_{0}}{R_{1}}\Theta \left(a-R_{1}\right)\int_{a-R_{1}}^{a+R_{1}}dRR\Phi \left(R\right)F_{2}^{\left(0\right)}\left(R\right)\left[a^{2}-{\left(R-R_{1}\right)}^{2}\right] \end{array}$$

If we choose in Expression (7), then we obtain Expression (6).

If the atom is located at the center of the droplet, then its interaction energy with the droplet will have the form:

(8) $$E_{a-d}\left(0,a\right)=4\pi n_{0}\int_{0}^{a}dRR^{2}\Phi \left(R\right)F_{2}^{\left(0\right)}\left(R\right).$$

In a boundary case, when the droplet radius approaches infinity, from (8), the expression for the atom interaction energy with unbounded liquid can be obtained:

(9) $$E_{a}=4\pi n_{0}\int_{0}^{\infty }dRR^{2}\Phi \left(R\right)F_{2}^{\left(0\right)}\left(R\right).$$

Expressions (8) and (9) have an explicit geometrical meaning.

The expression for the interaction energy of the atom with a semi-bounded liquid in the case of arbitrary distances from the atom to the surface, when it is important that the pair atom–atom correlations in the semi-bounded liquid are accounted for, can be derived from Expression (5) by means of transition $a\to \infty ,R_{1}-a=d=const$:

(10) $$E_{a-f}\left(d\right)=2\pi n_{0}\int_{d}^{\infty }dRF_{2}^{\left(0\right)}\left(R\right)\Phi \left(R\right)R\left(R-d\right)$$

where d is a distance from the atom to the surface.

The interaction energy of an atom that is located on a flat surface of a semi-bounded liquid in the case of the Fowler approximation will take a form of a particular case of (10):

(11) $$E_{a-f}\left(0\right)=2\pi n_{0}\int_{0}^{\infty }dRR^{2}F_{2}^{\left(0\right)}\left(R\right)\Phi \left(R\right).$$

In contrast to the results of continual approximation, the interaction energy (11) is limited. In Expressions (4)–(11) for the interaction energy, the divergence of the corresponding integrals in the accounting for the paired interatomic correlations is absent.

Let us consider a boundary case of atom interaction energy with a nanodroplet of liquid in a continual approximation. In the case of distances $R_{1}-a\gg \sigma$, where σ is a characteristic length for the pair distribution function, we can assume $F_{2}^{\left(0\right)}≅1$ and integrate using the explicit expression for the atom’s paired interaction potential. Thus, in the case of Lennard–Jones potential:

(12) $$\Phi \left(R\right)=4\varepsilon \left[{\left(\frac{\sigma }{R}\right)}^{12}-{\left(\frac{\sigma }{R}\right)}^{6}\right]$$

with the parameters σ and ε, we obtain:

(13) $$\begin{array}{c} E_{a-d}(a,R_{1})=\frac{4\pi n_{0}\varepsilon \sigma^{6}}{R_{1}}\{\frac{\left(R_{1}^{2}-a^{2}\right)\sigma^{6}}{10}\left[\frac{1}{{\left(R_{1}+a\right)}^{10}}-\frac{1}{{\left(R_{1}-a\right)}^{10}}\right]+\\ +\frac{1}{8}\sigma^{6}\left[\frac{1}{{\left(R_{1}+a\right)}^{8}}-\frac{1}{{\left(R_{1}-a\right)}^{8}}\right]-\frac{2}{9}R_{1}\sigma^{6}\left[\frac{1}{{\left(R_{1}+a\right)}^{9}}-\frac{1}{{\left(R_{1}-a\right)}^{9}}\right]+\\ +\frac{\left(a^{2}-R_{1}^{2}\right)}{4}\left[\frac{1}{{\left(R_{1}+a\right)}^{4}}-\frac{1}{{\left(R_{1}-a\right)}^{4}}\right]-\frac{1}{2}\left[\frac{1}{{\left(R_{1}+a\right)}^{2}}-\frac{1}{{\left(R_{1}-a\right)}^{2}}\right]+\\ +\frac{2}{3}R_{1}\left[\frac{1}{{\left(R_{1}+a\right)}^{3}}-\frac{1}{{\left(R_{1}-a\right)}^{3}}\right]\}. \end{array}$$

The interaction energy of an atom with the semi-infinite liquid in the boundary case a→∞ can be written as:

(14) $$E_{a-f}\left(d\right)=\frac{4\pi }{9}n_{0}\sigma^{3}\varepsilon \left\{\frac{1}{5}{\left(\frac{\sigma }{d}\right)}^{9}-\frac{3}{2}{\left(\frac{\sigma }{d}\right)}^{3}\right\},$$

where d is the distance between the atom and a flat surface. In the general case, the potential interaction parameters of an atom with a droplet and an atom with semi-infinite liquid are functions of density and temperature of the liquid. 

From Expression (14), we can obtain the position of the first zero $d_{0}$ of the potential and the value of the potential minimum \$d_{\min}$:

(15) $$d_{0}=\frac{\sigma }{\sqrt[{6}]{3}},d_{\min}=\sigma .$$

The depth of the potential well in which the atom near the flat surface moves will take the form:

(16) $$U=-\frac{8\pi n_{0}\sigma^{3}}{9}\varepsilon .$$

The interaction energy of an atom with a semi-infinite liquid at long distances from the surface is the inverse proportional cube of the distance to the surface and has the following asymptote:

(17) $$E_{a-f}≅-\frac{\pi n_{0}C}{6}\frac{1}{d^{3}},$$

where $C=4\varepsilon \sigma^{6}$ is a constant in the Van der Waals potential. Asymptotic behavior:

(18) $$E_{a-f}∼\frac{1}{d^{3}}$$

is obtained within the framework of macroscopic Van der Waals interaction theory (without accounting for delay effects) [20].

The numerical calculations were performed for the normal ${}^{4}He$ at a temperature T=2.2K and a density ρ=147 kg/m^3^, which corresponds to the density of the number of particles n=22.1266 nm^−3^. Parameters of the Lennard–Jones potential were chosen to be σ=2.576Å, ε=10.22K [13]. The pair distribution function was modeled by means of the distribution function obtained within the framework of the Barker–Henderson thermodynamic perturbation theory [10,16].

**Figure 2:**
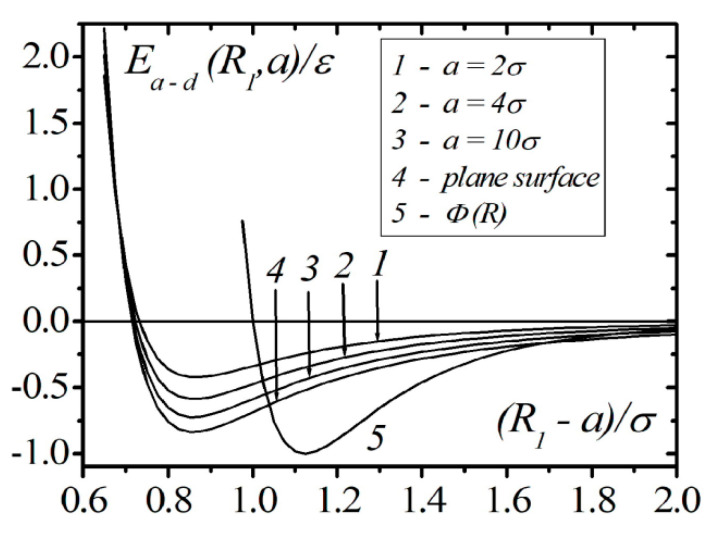
The results of model calculations of the energy of the interaction of atoms and nanodroplets of ${}^{4}He$ with different radiuses.

Figure 2 shows the results of calculations in continual approximation using Formula (13) for the interaction energy of an atom with a droplet of liquid helium for different values of droplet radius (curves 1–3) and a semi-bounded liquid (curve 4). The interaction energy of the pair of atoms, which is described by the Lennard–Jones potential (curve 5), is also shown. The calculations show that the position of the minimum of the interaction energy of an atom with droplets and semi-bounded liquids is significantly shifted to the shorter distances in comparison to the interatomic potential Φ(R). The depth of the potential well increases with the growth of the droplet radius and reaches saturation for the flat interface of the liquid. At a given density of the liquid, the depth of the potential well, even in the case of atom interaction with the flat interface, is less than in the case of atom–atom interaction. In the case of continual approximation, the interaction energy of the atom with its surroundings approaches infinity when an atom approaches a surface, i.e., the atom cannot reach the surface of the liquid. This result is not satisfactory for a liquid; however, to a certain level, it is acceptable for the modeling of atom interaction with a solid body, and it is used in absorption problems. The main disadvantage of the continuum approximation is complete neglect of the discrete surrounding structure effects and correlations between separate atoms with the surrounding atoms.

**Figure 3:**
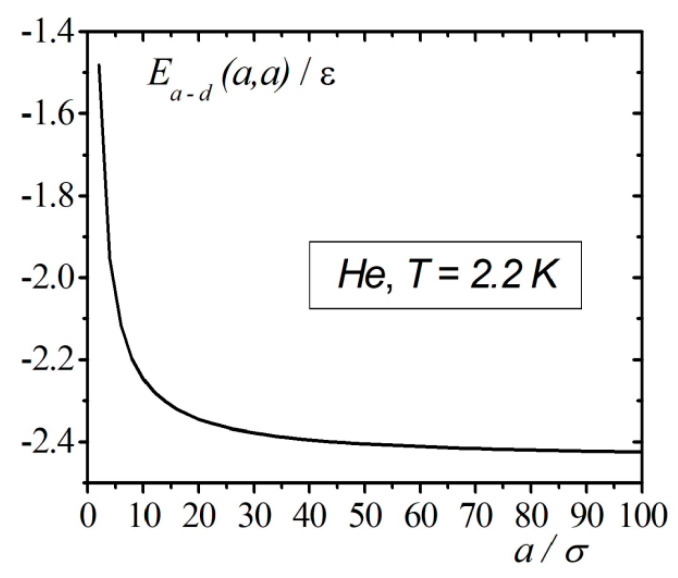
Results of calculations of the interaction energy of an atom with a droplet of ${}^{4}He$ when the atom is located on the surface of the droplet.

The calculation results of the interaction energy of an atom with a droplet in the framework of correlation theory in the case when the atom is located on the surface of the droplet are shown in Figure 3. From Figure 3, we can see that at droplet radius vales around [20−30σ], the energy $E_{a-d}\left(a,a\right)$, as a function of the radius, approaches the asymptotic value. In contrast to the continuum model, accounting for the interatomic correlations of the atom on the surface of the droplet demonstrates finite negative values of the energy, which corresponds to the attraction of the atom to the droplet.

**Figure 4:**
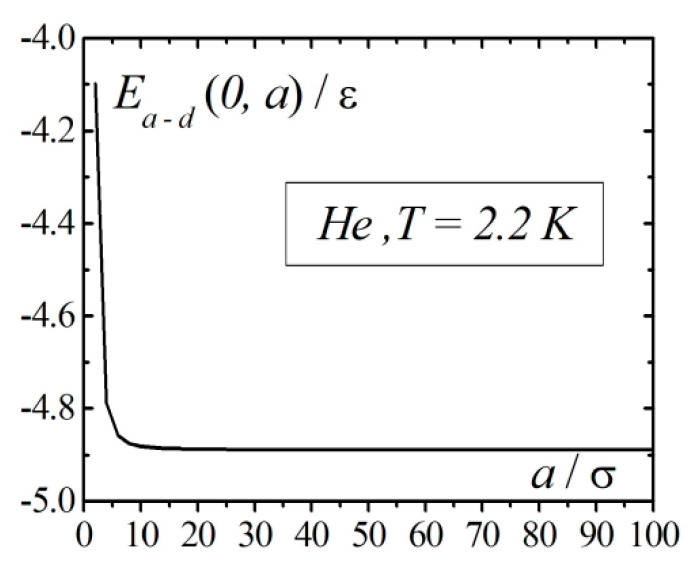
The interaction energy of the atom with a droplet as a function of the droplet radius for the case when the atom is located in the center of the droplet.

Figure 4 shows the calculation results of the interaction energy of an atom with a droplet as a function of the droplet radius $E_{a-d}\left(0,a\right)$, for the case when atom is located in the center of the droplet. From the graph in Figure 4, it is seen that at radius values of the order 10σ, the interaction energy of the atom with droplet $E_{a-d}\left(0,a\right)$ reaches its asymptotic values for the unbounded liquid; however, it remains finite.

**Figure 5:**
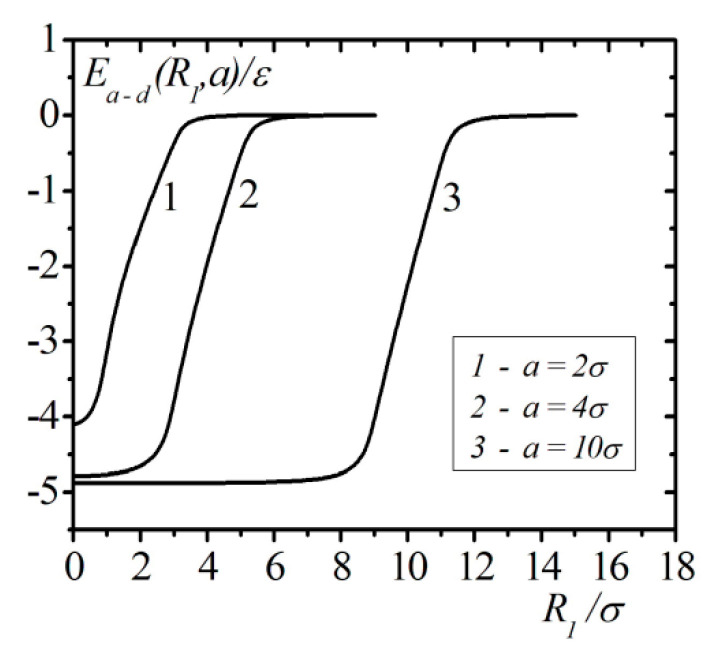
The interaction energy of the atom with the droplet of liquid ${}^{4}He$, accounting for correlation effects with variable droplet radius values.

Figure 5 shows the calculation results of the atom interaction energy with a liquid helium droplet, including accounting for the correlation effects at variable radius values and arbitrary distances from the atom to the center of the droplet. As can be seen from the graph, the interaction energy of an atom with a droplet exhibits a saturation effect. This effect can be seen in the following circumstances. (1) The droplet radius values are of the order of 4σ, and the number of atoms in the droplet is around 101. A one-atom potential is formed, which covers most of the droplet and corresponds to the asymptotic value for the unbounded liquid. (2) The thickness of the near-surface layer, where the interaction energy varies from its value inside the droplet to the asymptotic value outside the droplet, quickly reaches the values of the order 6σ at radius growth. These results indicate that a majority of atoms inside the droplet with a radius a>10σ are under the influence of self-consistent potential, which is similar to that of the homogeneous unbounded liquid, and the gradient of this potential is located at the near-surface layer of the thickness 6σ. As a result, at radius growth, the thickness of the atom density profile of the near-surface layer quickly reaches values corresponding to the flat surface.

**Figure 6:**
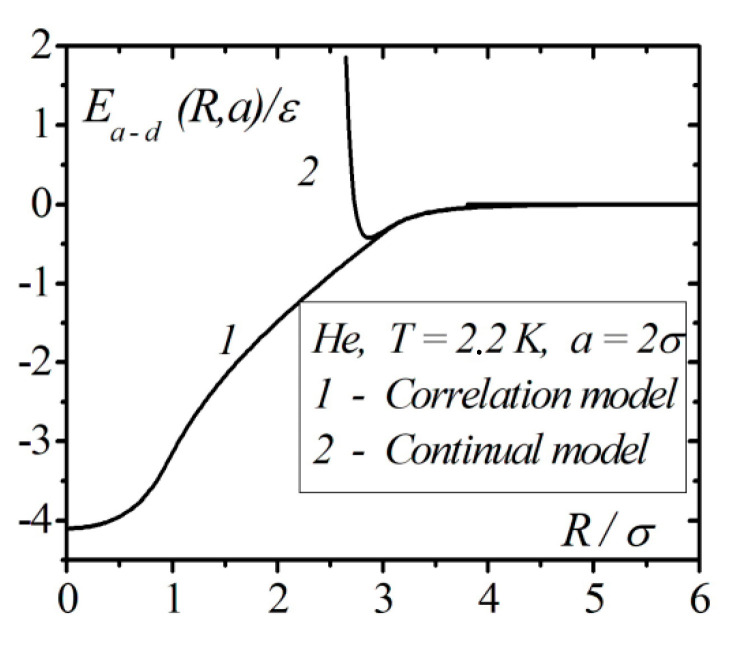
Comparison of the energies of interaction of an atom with a droplet of ${}^{4}He$ with radius a=2σ in the continual model and taking into account correlation effects.

For comparison, Figure 6 shows the dependences of the interaction energies of an atom and ${}^{4}He$ nanodroplet of radius 2σ in the continual model and taking into account the correlation effects. The two curves practically coincide only for large distances R>2.8σ. At smaller distances, there is a significant difference and in the boundary case R→2σ+0 in the continual model $E_{a\;-\;d}\;\left(R,a\right)\to \infty$.

## 3. Microscopic Theory of Nanodroplet Surface Energy in Fowler Approximation

Let us consider a simple liquid in a volume V, which is described by the Hamiltonian:

(19) $$H=\sum_{i=1}^{N}\frac{P_{i}^{2}}{2M}+\frac{1}{2}\sum_{i\neq j=1}^{N}\Phi \left(\left|R_{i}-R_{j}\right|\right),$$

where $P_{i},M$ are the impulse and mass of an atom, respectively; $\Phi \left(\left|R_{i}-R_{j}\right|\right)$ is the central–symmetric potential of interatomic interaction; and N is the number of atoms. After an averaging within the framework of the distribution function method of groups of particles [10,13,14,15,16,19], for the energy of liquid, we obtain:

(20) $$\begin{array}{c} E=\langle H\rangle =E_{k}+E_{p}=\frac{3}{2}Nk_{B}T+\\ +\frac{N\left(N-1\right)}{2V^{2}}\int_{V}d^{3}R_{1}\int_{V}d^{3}R_{2}F_{2}\left(\left|R_{1}-R_{2}\right|\right)\Phi \left(\left|R_{1}-R_{2}\right|\right) \end{array}$$

where V is the system volume and $k_{B}$ is the Boltzman constant.

We can divide the volume of the system into two parts $V=V_{1}+V_{2}$, where $V_{1}$ is the volume of the droplet and $V_{2}$\ is the volume of the liquid around the droplet. Then, the potential energy of the liquid can be written as follows:

(21) $$\begin{array}{c} E_{p}=\frac{n_{0}^{2}}{2}\int_{V_{1}+V_{2}}d^{3}R_{1}\int_{V_{1}+V_{2}}d^{3}R_{2}F_{2}\left(\left|R_{1}-R_{2}\right|\right)\Phi \left(\left|R_{1}-R_{2}\right|\right)=\\ =\frac{n_{0}^{2}}{2}\int_{V_{1}}d^{3}R_{1}\int_{V_{1}}d^{3}R_{2}F_{2}\left(\left|R_{1}-R_{2}\right|\right)\Phi \left(\left|R_{1}-R_{2}\right|\right)+\\ +2\frac{n_{0}^{2}}{2}\int_{V_{1}}d^{3}R_{1}\int_{V_{2}}d^{3}R_{2}F_{2}\left(\left|R_{1}-R_{2}\right|\right)\Phi \left(\left|R_{1}-R_{2}\right|\right)+\\ +\frac{n_{0}^{2}}{2}\int_{V_{2}}d^{3}R_{1}\int_{V_{2}}d^{3}R_{2}F_{2}\left(\left|R_{1}-R_{2}\right|\right)\Phi \left(\left|R_{1}-R_{2}\right|\right), \end{array}$$

where the first term is the bulk component of the droplet energy, the second term is the interaction energy of molecules inside the droplet with the molecules located outside, and the third term is the molecules’ interaction energy in the liquid with the spherical pore. The first and the third terms are proportional to the volumes of the liquid droplet and liquid with the pore, correspondingly, and the second term is proportional to the surface area of the droplet.

Let us split the system into the droplet of radius a and the remainder of the volume (liquid with a pore of radius a). The potential energy $E^{\prime }$ of the separated system is written:

(22) $$\begin{array}{c} E^{\prime }=\frac{n_{0}^{2}}{2}\int_{V_{1}}d^{3}R_{1}\int_{V_{1}}d^{3}R_{2}F_{2}\left(\left|R_{1}-R_{2}\right|\right)\Phi \left(\left|R_{1}-R_{2}\right|\right)+\\ +\frac{n_{0}^{2}}{2}\int_{V_{2}}d^{3}R_{1}\int_{V_{2}}d^{3}R_{2}F_{2}\left(\left|R_{1}-R_{2}\right|\right)\Phi \left(\left|R_{1}-R_{2}\right|\right), \end{array}$$

where the first term is the energy of the droplet, and the second term is the energy of the liquid with a pore. The potential energy of the system separation is $E^{\prime }-E_{p}$. The energy of the system separation is proportional to the surface area of the sphere. The specific separation energy per unit of the formed surface $S=4\pi a^{2}$ can be written as:

(23) $$\sigma =\frac{1}{2}\left(E^{\prime }-E_{p}\right)=-\frac{n_{0}^{2}}{2}\int_{V_{1}}d^{3}R_{1}\int_{V_{2}}d^{3}R_{2}F_{2}\left(\left|R_{1}-R_{2}\right|\right)\Phi \left(\left|R_{1}-R_{2}\right|\right).$$

The specific separation energy (23) corresponds to the previously derived surface energy of the droplet and pore in Fowler’s approximation [16,19]. Fowler’s approximation corresponds to the “step” form of the molecules’ density profiles on the boundary of the droplet and pore inside the liquid, with pair distribution functions similar to that of the homogeneous liquid.

The surface energy of the droplet can be defined as the difference between the total energy of the droplet and the energy of the homogeneous phase in the volume corresponding to the volume of the droplet divided by the surface area of the droplet. This definition is equivalent to distinguishing the volumetric and surface parts in the total energy of the droplet. For the model calculations, the pair distribution function was approximated as follows:

(24) $$F_{2}\left(R_{1},R_{2}\right)≅\Theta \left(a-R_{1}\right)\Theta \left(a-R_{2}\right)F_{2}^{\left(0\right)}\left(\left|R_{1}-R_{2}\right|\right),$$

where $F_{2}^{\left(0\right)}\left(\left|R_{1}-R_{2}\right|\right)$ is the pair distribution function of atoms in a homogeneous liquid; and a is the droplet radius. Approximation (24) is similar to the Kirkwood–Buff approximation for a semi-bounded liquid with a flat interface [16,19,21].

The received expressions for the droplet energy, pore energy, and separation energy of a liquid into a droplet and a liquid with pore can be integrated in spherical coordinates. Thus, using Approximation (24), the energy of the liquid droplet can be written as:

(25) $$E_{drop}=\frac{3}{2}Nk_{B}T+\frac{4}{3}\pi a^{3}\varepsilon +\sigma S,$$

where

(26) $$\varepsilon =2\pi n_{0}^{2}\int_{0}^{\infty }dRR^{2}F_{2}^{\left(0\right)}\left(R\right)\Phi \left(R\right)$$

is the bulk density of the potential energy of a liquid; $n_{0}$ is the density of the number of particles in a homogeneous liquid; and S is the surface area of the droplet.

The surface energy of the droplet is defined as:

(27) $$\sigma =\sigma_{0}+\Delta \sigma ,$$

where

(28) $$\sigma_{0}=-\frac{\pi n_{0}^{2}}{2}\int_{0}^{\infty }dRR^{3}F_{2}^{\left(0\right)}\left(R\right)\Phi \left(R\right)$$

is the surface energy of the flat interface of the liquid in Fowler’s approximation [16,19,21,22].

(29) $$\begin{array}{c} \Delta \sigma =\frac{\pi n_{0}^{2}}{2}\int_{2a}^{\infty }dRR^{3}\Phi \left(R\right)F_{2}^{\left(0\right)}\left(R\right)+\frac{\pi n_{0}^{2}}{24a^{2}}\int_{0}^{2a}dRR^{5}\Phi \left(R\right)F_{2}^{\left(0\right)}\left(R\right)-\\ -\frac{\pi n_{0}^{2}}{2}\cdot \frac{4\pi a}{3}\int_{2a}^{\infty }dRR^{2}\Phi \left(R\right)F_{2}^{\left(0\right)}\left(R\right) \end{array}$$

is an additional term for the surface energy of the droplet due to the surface curvature. In the boundary case of the large droplet radius values $\underset{a\to \infty }{\lim}\sigma =\sigma_{0}$, this corresponds to the surface energy of the liquid with a flat interface. For small values of the droplet radius, $\underset{a\to 0}{\lim}\sigma =0.$

**Figure 7:**
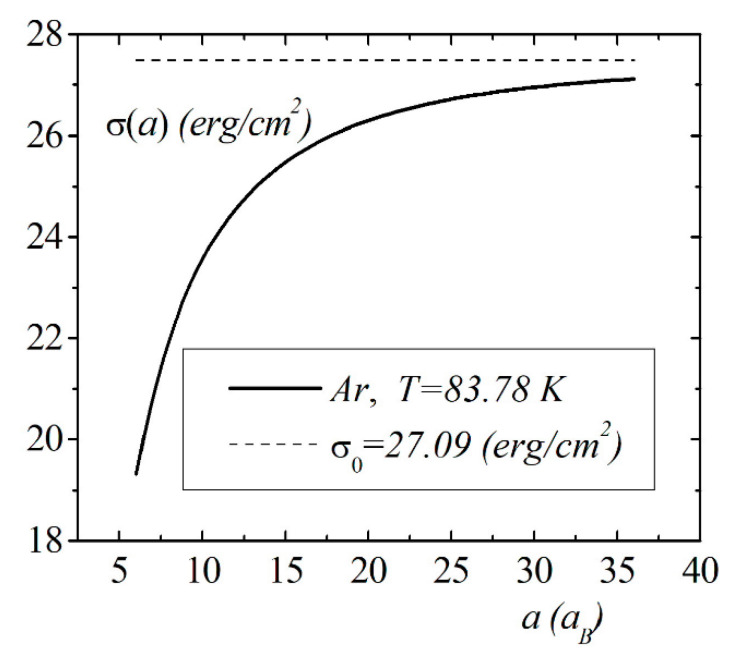
The size dependence of the surface energy σ(a) for the Ar droplet.

The model calculations of the size dependence of the surface energy of the droplet σ(a) were performed for simple liquids using the Lennard–Jones potential and pair distribution function, which were obtained within the framework of the Wicks–Chandler–Anderson (WCA) thermodynamic perturbation theory [16,22,23,24]. Figure 7 shows the calculation results for the dependence σ(a) of argon at the melting point. The atoms’ pair distribution function was calculated according to the WCA procedure. The Lennard–Jones parameters were chosen as $\varepsilon =124K,\sigma =3.418\overset{0}{A}$, and the droplet radius was depicted in terms of Bohr radius $a_{B}$. The asymptotic value of the surface energy at a→∞ is equal $\sigma_{0}=27.04$ erg/cm^2^.

**Figure 8:**
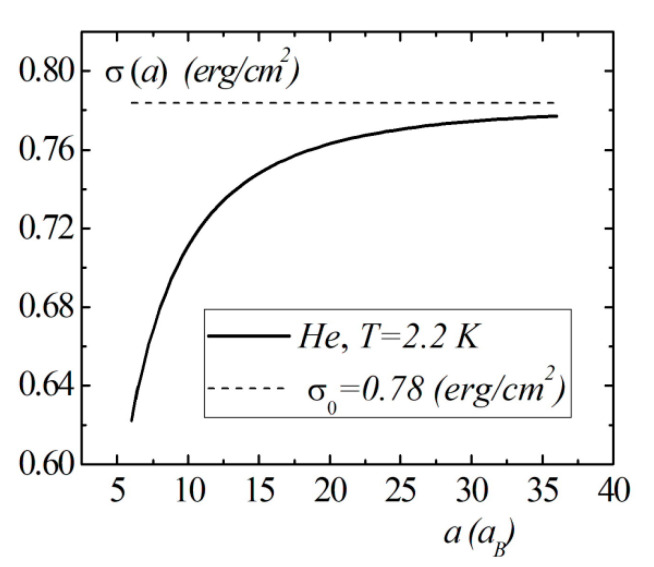
The size dependence of the surface energy σ(a) for the He droplet.

**Figure 9:**
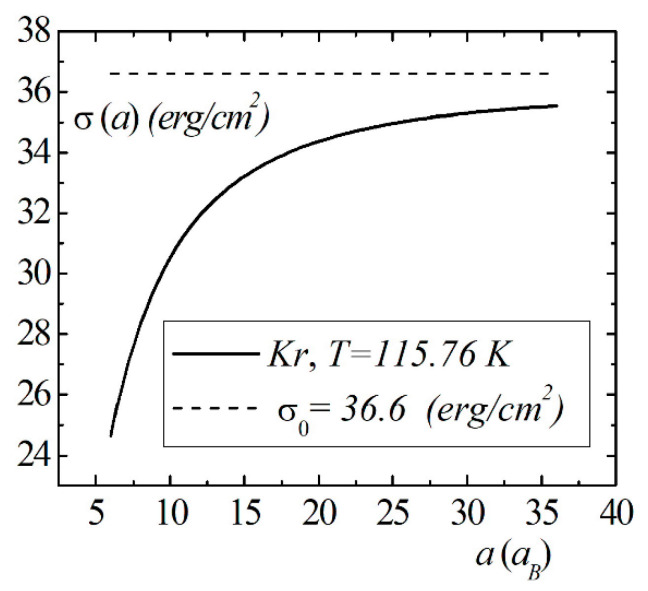
The size dependence of the surface energy σ(a) for the Kr droplet.

**Figure 10:**
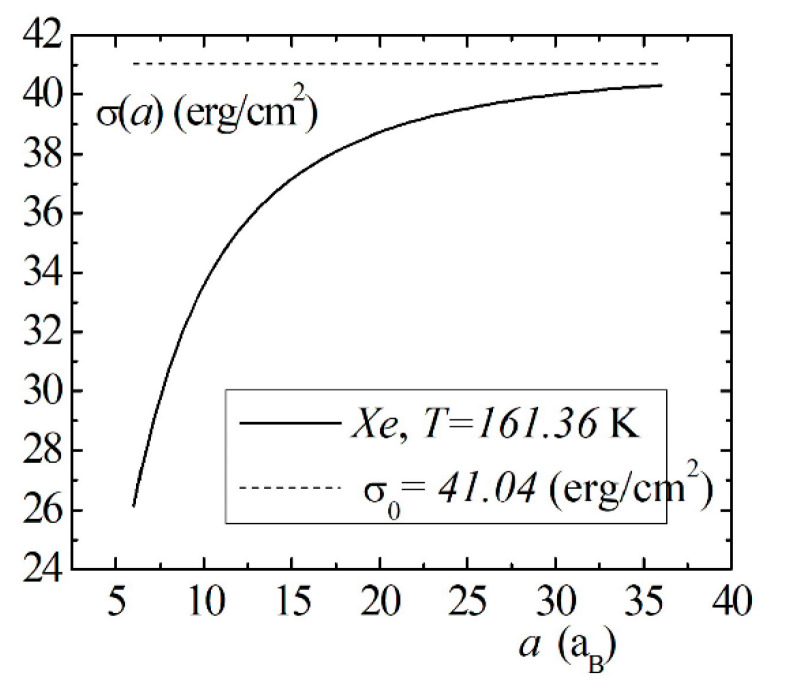
The size dependence of the surface energy σ(a) for the Xe droplet.

Model calculations of the dimensional dependence of the surface energy of gases He, Kr, Xe are presented in Figure 8, Figure 9 and Figure 10. The parameters of Lennard–Jones potentials are taken from a monograph [13]. Model calculations for all elements are performed for temperatures and densities corresponding to triple points. Note the similarity of the dimensional dependences of σ(a) for the selected group of elements. The surface energy shows a strong dependence for nanosized droplets. With an increase in size, the surface energy of nanodroplet approaches the value of the surface energy of a flat surface $\sigma_{0}$.

When a droplet of liquid is located in a gas, then for the surface energy of the boundary of the droplet–gas interface, we use a representation in which the surface energy of the gas phase is described as the surface energy of a gas with a pore. We assume that the density of the number of particles in liquid is $n_{0}$, and in gas, it is $n_{1}$. The pair distribution functions of particles in a homogeneous liquid and gas are $F_{20}$ and $F_{21}$, respectively. Then, the surface energy of the spherical liquid–gas interface, $\sigma_{l-g}$, is reduced in comparison to the surface energy of the liquid droplet in vacuum to a value that is equal to the surface energy of the pore with a vacuum in the gas

(30) $$\begin{array}{c} \sigma_{l-g}=\sigma_{0l}-\sigma_{0g}+\frac{\pi n_{0}^{2}}{2}[\int_{2a}^{\infty }dRR^{3}F_{20}^{\left(0\right)}\left(R\right)\Phi \left(R\right)-\\ -\frac{4a}{3}\int_{2a}^{\infty }dRR^{2}F_{20}^{\left(0\right)}\left(R\right)\Phi \left(R\right)+\frac{1}{12a^{2}}\int_{0}^{2a}dRR^{5}\Phi \left(R\right)F_{20}^{\left(0\right)}\left(R\right)]-\\ +\frac{\pi n_{1}^{2}}{2}[\int_{2a}^{\infty }dRR^{3}F_{21}^{\left(0\right)}\left(R\right)\Phi \left(R\right)-\frac{4a}{3}\int_{2a}^{\infty }dRR^{2}F_{21}^{\left(0\right)}\left(R\right)\Phi \left(R\right)+\\ +\frac{1}{12a^{2}}\int_{0}^{2a}dRR^{5}F_{21}^{\left(0\right)}\left(R\right)\Phi \left(R\right)] \end{array}$$

where

(31) $$\sigma_{ol}=-\frac{\pi n_{0}^{2}}{2}\int_{0}^{\infty }dRR^{3}F_{20}^{\left(0\right)}\left(R\right)\Phi \left(R\right)$$

is the surface energy of the flat liquid–vacuum interface; and

(32) $$\sigma_{og}=-\frac{\pi n_{1}^{2}}{2}\int_{0}^{\infty }dRR^{3}F_{21}^{\left(0\right)}\left(R\right)\Phi \left(R\right)$$

is the surface energy of the flat gas–vacuum interface.

In the boundary case of gas density growth, $n_{1}\to n_{0}$, the surface energy of the spherical liquid–gas interface approaches zero, $\sigma_{l-g}\to 0$. In the boundary case when a→∞, we obtain $\underset{a\to \infty }{\lim}\sigma_{l-g}=\sigma_{0l}-\sigma_{0g}.$

In this section, within the framework of the correlation theory of inhomogeneous liquids, the general expressions for the bulk and surface terms in the droplet energy as a function of the radius were obtained. Using the Fowler approximation, we were able to reduce all of the terms to the single integrals, which significantly simplified the model calculations of the size dependence. The size dependence of the surface energy was calculated for spherical droplets of the simple dielectric liquids as a function of the radius in Fowler’s approximations. In the boundary case of the large radius of the droplet, the surface energy approaches the value for the flat surface. The strong size dependence of the droplet surface energy is observed at nanometer scales of the droplet radius. When the droplet radius $a<15a_{B}$, a significant decrease in the surface energy is observed.

The derived approach is used to calculate the surface energy of two-phase system of nanodroplet–gas, including accounting for the paired interparticle interactions and correlations. In the vicinity of the mixing point of liquid and gas, the surface energy of the nanodroplet in gas approaches zero. The size dependence of the nanodroplet surface energy in gas is similar to the size dependence of the nanodroplet in vacuum.

## 4. The Correlation Theory of Interaction Energy between Two Nanodroplets and Two Nano-Pores in Liquid

The contemporary state of development in molecular–kinetic representations of the interaction of macroscopic bodies by means of intermolecular forces is described in the monograph [11]. In [11], the expressions for the interaction energy of the bodies with different geometry were derived, and these used the attractive potential of intermolecular interaction. This potential corresponds to the Van der Waals potential, and the repulsive intermolecular forces and the interparticle correlations in these calculations are not taken into account. Thus, the obtained results in [11] for the interaction energies of macroscopic bodies correspond to the asymptotic values for large distances. Taking into account the short-range intermolecular forces requires a theory that also takes into consideration the intermolecular correlations. This approach can be implemented within the framework of the statistical theory of multiparticle systems [10,13,14,15,16]. The kinetics of air dispersal and cavitation systems requires knowledge of the energy of the interaction processes of nanodroplets and nanopores as well as that in their ensembles with the surrounding phase.

In the current section, we established the theory of the interaction energy for two droplets of a simple liquid within the framework of the distribution function method of the groups of particles. The derived expressions for the interaction energy of two nanodroplets take into account paired interparticle interactions and correlations, and they are applicable for the arbitrary distances between nanodroplets or nanopores.

We postulate in a homogeneous liquid that occupies a volume V a presence of two pores of volumes $V_{1}$ and $V_{2}$. The volume of the liquid without the volume of two pores we denote $V^{\prime }$. The potential energy of the liquid with two pores can be written:

(33) $$E=\frac{1}{2}n_{0}^{2}\int_{V^{\prime }}d^{3}r_{1}\int_{V^{\prime }}d^{3}r_{2}\Phi \left(\left|r_{1}-r_{2}\right|\right)F_{2}\left(\left|r_{1}-r_{2}\right|\right),$$

where $\Phi \left(\left|r_{1}-r_{2}\right|\right)$ is the potential energy of interaction of two atoms; $F_{2}\left(\left|r_{1}-r_{2}\right|\right)$ is the pair distribution function of atoms in a homogeneous liquid; and $n_{0}=N/V$ is the density of the number of particles in a homogeneous liquid. 

From the energy in (33), we can separate the part that corresponds to the energy of a homogeneous liquid in a volume V:

(34) $$\begin{array}{c} E=\frac{1}{2}n_{0}^{2}\left(\int_{V^{\prime }}d^{3}r_{1}+\int_{V_{1}}d^{3}r_{1}+\int_{V_{2}}d^{3}r_{1}-\int_{V_{1}}d^{3}r_{1}-\int_{V_{2}}d^{3}r_{1}\right)\times \\ \times \left(\int_{V^{\prime }}d^{3}r_{2}+\int_{V_{1}}d^{3}r_{2}+\int_{V_{2}}d^{3}r_{2}-\int_{V_{1}}d^{3}r_{2}-\int_{V_{2}}d^{3}r_{2}\right)\Phi \left(\left|r_{1}-r_{2}\right|\right)F_{2}\left(\left|r_{1}-r_{2}\right|\right)=\\ =\frac{1}{2}n_{0}^{2}\left(\int_{V}d^{3}r_{1}-\int_{V_{1}}d^{3}r_{1}-\int_{V_{2}}d^{3}r_{1}\right)\left(\int_{V}d^{3}r_{2}-\int_{V_{1}}d^{3}r_{2}-\int_{V_{2}}d^{3}r_{2}\right)\times \\ \times \Phi \left(\left|r_{1}-r_{2}\right|\right)F_{2}\left(\left|r_{1}-r_{2}\right|\right). \end{array}$$

The energy Expression (34) contains nine terms whose physical meaning we clarify in the following. The first term is the potential energy of a homogeneous liquid in a volume V:

(35) $$\frac{1}{2}n_{0}^{2}\int_{V}d^{3}r_{1}\int_{V}d^{3}r_{2}\Phi \left(\left|r_{1}-r_{2}\right|\right)F_{2}\left(\left|r_{1}-r_{2}\right|\right)=\varepsilon V.$$

This energy (35) can be represented as the product of the bulk density of the potential energy ε and the volume of the system V. The sum of the contributions from (34):

(36) $$\begin{array}{c} -\frac{1}{2}n_{0}^{2}\int_{V}d^{3}r_{1}\int_{V_{1}}d^{3}r_{2}\Phi \left(\left|r_{1}-r_{2}\right|\right)F_{2}\left(\left|r_{1}-r_{2}\right|\right)+\\ +\frac{1}{2}n_{0}^{2}\int_{V_{1}}d^{3}r_{1}\int_{V_{1}}d^{3}r_{2}\Phi \left(\left|r_{1}-r_{2}\right|\right)F_{2}\left(\left|r_{1}-r_{2}\right|\right)=\\ =-\frac{1}{2}n_{0}^{2}\left(\int_{V}d^{3}r_{1}-\int_{V_{1}}d^{3}r_{1}\right)\int_{V_{1}}d^{3}r_{2}\Phi \left(\left|r_{1}-r_{2}\right|\right)F_{2}\left(\left|r_{1}-r_{2}\right|\right)=-\sigma_{1}S_{1}, \end{array}$$

corresponds to the potential energy of atoms’ interaction inside the volume $V_{1}$ with the atoms surrounding the first pore volume $V-V_{1}$, which is proportional to the surface area of the first pore $S_{1}$ and the surface energy of the first pore $\sigma_{1}$.

The next sum of the two contributions in (34) can be written as:

(37) $$\begin{array}{c} -\frac{1}{2}n_{0}^{2}\int_{V}d^{3}r_{1}\int_{V_{2}}d^{3}r_{2}\Phi \left(\left|r_{1}-r_{2}\right|\right)F_{2}\left(\left|r_{1}-r_{2}\right|\right)+\\ +\frac{1}{2}n_{0}^{2}\int_{V_{2}}d^{3}r_{1}\int_{V_{2}}d^{3}r_{2}\Phi \left(\left|r_{1}-r_{2}\right|\right)F_{2}\left(\left|r_{1}-r_{2}\right|\right)=\\ =-\frac{1}{2}n_{0}^{2}\left(\int_{V}d^{3}r_{1}-\int_{V_{2}}d^{3}r_{1}\right)\int_{V_{2}}d^{3}r_{2}\Phi \left(\left|r_{1}-r_{2}\right|\right)F_{2}\left(\left|r_{1}-r_{2}\right|\right)=-\sigma_{2}S_{2}, \end{array}$$

which corresponds to the potential interaction energy of atoms inside the volume $V_{2}$ and atoms surrounding the second pore volume $V-V_{2}$. This term is proportional to the surface area of the second pore $S_{2}$ and to the surface energy of the second pore $\sigma_{2}$.

The sum of the following two terms:

(38) $$\begin{array}{c} +\frac{1}{2}n_{0}^{2}\int_{V_{1}}d^{3}r_{1}\int_{V_{2}}d^{3}r_{2}\Phi \left(\left|r_{1}-r_{2}\right|\right)F_{2}\left(\left|r_{1}-r_{2}\right|\right)+\\ +\frac{1}{2}n_{0}^{2}\int_{V_{2}}d^{3}r_{1}\int_{V_{1}}d^{3}r_{2}\Phi \left(\left|r_{1}-r_{2}\right|\right)F_{2}\left(\left|r_{1}-r_{2}\right|\right) \end{array}$$

corresponds to the duplicated interaction energy of two droplets with volumes $V_{1}$ and $V_{2}$.

For the sum of the contributions in (34), which are not accounted for in Expressions (36)–(38), we denote ΔE and write in the form:

(39) $$\begin{array}{c} \Delta E=-\frac{1}{2}n_{0}^{2}\int_{V_{1}}d^{3}r_{1}\int_{V}d^{3}r_{2}\Phi \left(\left|r_{1}-r_{2}\right|\right)F_{2}\left(\left|r_{1}-r_{2}\right|\right)+\\ +\frac{1}{2}n_{0}^{2}\int_{V_{1}}d^{3}r_{1}\int_{V_{2}}d^{3}r_{2}\Phi \left(\left|r_{1}-r_{2}\right|\right)F_{2}\left(\left|r_{1}-r_{2}\right|\right)+\\ -\frac{1}{2}n_{0}^{2}\int_{V_{2}}d^{3}r_{1}\int_{V}d^{3}r_{2}\Phi \left(\left|r_{1}-r_{2}\right|\right)F_{2}\left(\left|r_{1}-r_{2}\right|\right)+\\ +\frac{1}{2}n_{0}^{2}\int_{V_{2}}d^{3}r_{1}\int_{V_{1}}d^{3}r_{2}\Phi \left(\left|r_{1}-r_{2}\right|\right)F_{2}\left(\left|r_{1}-r_{2}\right|\right)=\\ =-\frac{1}{2}n_{0}^{2}\int_{V_{1}}d^{3}r_{1}\left(\int_{V}d^{3}r_{2}-\int_{V_{2}}d^{3}r_{2}\right)\Phi \left(\left|r_{1}-r_{2}\right|\right)F_{2}\left(\left|r_{1}-r_{2}\right|\right)-\\ -\frac{1}{2}n_{0}^{2}\int_{V_{2}}d^{3}r_{1}\left(\int_{V}d^{3}r_{2}-\int_{V_{1}}d^{3}r_{2}\right)\Phi \left(\left|r_{1}-r_{2}\right|\right)F_{2}\left(\left|r_{1}-r_{2}\right|\right)=\\ =-\frac{1}{2}n_{0}^{2}\int_{V_{1}}d^{3}r_{1}\int_{V-V_{2}}d^{3}r_{2}\Phi \left(\left|r_{1}-r_{2}\right|\right)F_{2}\left(\left|r_{1}-r_{2}\right|\right)-\\ -\frac{1}{2}n_{0}^{2}\int_{V_{2}}d^{3}r_{1}\int_{V-V_{1}}d^{3}r_{2}\Phi \left(\left|r_{1}-r_{2}\right|\right)F_{2}\left(\left|r_{1}-r_{2}\right|\right). \end{array}$$

Simplifying (39)

(40) $$\begin{array}{c} \Delta E=-\frac{1}{2}n_{0}^{2}\int_{V_{1}}d^{3}r_{1}\left(\int_{V-V_{1}-V_{2}}d^{3}r_{2}+\int_{V_{1}}d^{3}r_{2}\right)\Phi \left(\left|r_{1}-r_{2}\right|\right)F_{2}\left(\left|r_{1}-r_{2}\right|\right)-\\ -\frac{1}{2}n_{0}^{2}\int_{V_{2}}d^{3}r_{1}\left(\int_{V-V_{1}-V_{2}}d^{3}r_{2}+\int_{V_{2}}d^{3}r_{2}\right)\Phi \left(\left|r_{1}-r_{2}\right|\right)F_{2}\left(\left|r_{1}-r_{2}\right|\right), \end{array}$$

we get

(41) $$\begin{array}{c} \Delta E=-\frac{1}{2}n_{0}^{2}\int_{V_{1}}d^{3}r_{1}\int_{V_{1}}d^{3}r_{2}\Phi \left(\left|r_{1}-r_{2}\right|\right)F_{2}\left(\left|r_{1}-r_{2}\right|\right)-\\ -\frac{1}{2}n_{0}^{2}\int_{V_{1}}d^{3}r_{1}\int_{V-V_{1}-V_{2}}d^{3}r_{2}\Phi \left(\left|r_{1}-r_{2}\right|\right)F_{2}\left(\left|r_{1}-r_{2}\right|\right)-\\ -\frac{1}{2}n_{0}^{2}\int_{V_{2}}d^{3}r_{1}\int_{V_{2}}d^{3}r_{2}\Phi \left(\left|r_{1}-r_{2}\right|\right)F_{2}\left(\left|r_{1}-r_{2}\right|\right)-\\ -\frac{1}{2}n_{0}^{2}\int_{V-V_{2}-V_{1}}d^{3}r_{1}\int_{V_{2}}d^{3}r_{2}\Phi \left(\left|r_{1}-r_{2}\right|\right)F_{2}\left(\left|r_{1}-r_{2}\right|\right). \end{array}$$

The first term in Expression (41) corresponds to the potential energy of liquid in the volume $V_{1}$ and contains the bulk contribution $-\varepsilon V_{1}$. Similarly, the third term in (41) contains a bulk part $-\varepsilon V_{2}$ of the potential energy of a liquid in a volume $V_{2}$. Thus, (41) takes the form:

(42) $$\begin{array}{c} \Delta E=-\varepsilon \cdot V_{1}-\varepsilon \cdot V_{2}-\\ -\frac{1}{2}n_{0}^{2}\int_{V_{1}}d^{3}r_{1}\int_{V-V_{1}-V_{2}}d^{3}r_{2}\Phi \left(\left|r_{1}-r_{2}\right|\right)F_{2}\left(\left|r_{1}-r_{2}\right|\right)-\\ -\frac{1}{2}n_{0}^{2}\int_{V-V_{2}-V_{1}}d^{3}r_{1}\int_{V_{2}}d^{3}r_{2}\Phi \left(\left|r_{1}-r_{2}\right|\right)F_{2}\left(\left|r_{1}-r_{2}\right|\right). \end{array}$$

The potential energy of the first and second droplets is $E_{1drop}=\varepsilon \cdot V_{1}+\sigma_{1}\cdot S_{1}$ and $E_{2drop}=\varepsilon \cdot V_{2}+\sigma_{2}\cdot S_{2}$, respectively.

Then, the potential energy of the liquid with two pores (33) will take the form:

(43) $$\begin{array}{c} E=\frac{1}{2}n_{0}^{2}\int_{V^{\prime }}d^{3}r_{1}\int_{V^{\prime }}d^{3}r_{2}\Phi \left(\left|r_{1}-r_{2}\right|\right)F_{2}\left(\left|r_{1}-r_{2}\right|\right)=\\ =\varepsilon \cdot V+\sigma_{1}\cdot S_{1}+\sigma_{2}\cdot S_{2}-E_{1drop}-E_{2drop}+E_{1}+E_{2}, \end{array}$$

where:

(44) $$E_{1}=-\frac{1}{2}n_{0}^{2}\int_{V_{1}}d^{3}r_{1}\int_{V-V_{1}-V_{2}}d^{3}r_{2}\Phi \left(\left|r_{1}-r_{2}\right|\right)F_{2}\left(\left|r_{1}-r_{2}\right|\right),$$

(45) $$E_{2}=-\frac{1}{2}n_{0}^{2}\int_{V-V_{2}-V_{1}}d^{3}r_{1}\int_{V_{2}}d^{3}r_{2}\Phi \left(\left|r_{1}-r_{2}\right|\right)F_{2}\left(\left|r_{1}-r_{2}\right|\right).$$

The energy expressions of (44) and (45) can be rewritten as:

(46) $$\begin{array}{c} E_{1}=-\frac{1}{2}n_{0}^{2}\int_{V_{1}}d^{3}r_{1}\int_{V-V_{1}}d^{3}r_{2}\Phi \left(\left|r_{1}-r_{2}\right|\right)F_{2}\left(\left|r_{1}-r_{2}\right|\right)+\\ +\frac{1}{2}n_{0}^{2}\int_{V_{1}}d^{3}r_{1}\int_{V_{2}}d^{3}r_{2}\Phi \left(\left|r_{1}-r_{2}\right|\right)F_{2}\left(\left|r_{1}-r_{2}\right|\right)=\\ =-\sigma_{1}\cdot S_{1}+\frac{1}{2}n_{0}^{2}\int_{V_{1}}d^{3}r_{1}\int_{V_{2}}d^{3}r_{2}\Phi \left(\left|r_{1}-r_{2}\right|\right)F_{2}\left(\left|r_{1}-r_{2}\right|\right). \end{array}$$

(47) $$\begin{array}{c} E_{2}=-\frac{1}{2}n_{0}^{2}\int_{V-V_{2}}d^{3}r_{1}\int_{V_{2}}d^{3}r_{2}\Phi \left(\left|r_{1}-r_{2}\right|\right)F_{2}\left(\left|r_{1}-r_{2}\right|\right)+\\ +\frac{1}{2}n_{0}^{2}\int_{V_{1}}d^{3}r_{1}\int_{V_{2}}d^{3}r_{2}\Phi \left(\left|r_{1}-r_{2}\right|\right)F_{2}\left(\left|r_{1}-r_{2}\right|\right)=\\ =-\sigma_{2}\cdot S_{2}+\frac{1}{2}n_{0}^{2}\int_{V_{1}}d^{3}r_{1}\int_{V_{2}}d^{3}r_{2}\Phi \left(\left|r_{1}-r_{2}\right|\right)F_{2}\left(\left|r_{1}-r_{2}\right|\right). \end{array}$$

where the second terms in (46) and (47) correspond to the potential interaction energy of two droplets or two pores:

(48) $$E_{drop-drop}=E_{pore-pore}=\frac{1}{2}n_{0}^{2}\int_{V_{1}}d^{3}r_{1}\int_{V_{2}}d^{3}r_{2}\Phi \left(\left|r_{1}-r_{2}\right|\right)F_{2}\left(\left|r_{1}-r_{2}\right|\right).$$

Substituting Expressions (46)–(48) into (43):

(49) $$\begin{array}{c} E=\varepsilon \cdot V+\sigma_{1}\cdot S_{1}+\sigma_{2}\cdot S_{2}-E_{1drop}-E_{2drop}-\\ -\sigma_{1}\cdot S_{1}-\sigma_{2}\cdot S_{2}+E_{drop-drop}+E_{pore-pore}=\\ =\varepsilon \cdot V-E_{1drop}-E_{2drop}+E_{drop-drop}+E_{pore-pore}, \end{array}$$

we receive the expression for the potential energy of homogeneous liquid in a volume V

(50) $$\varepsilon \cdot V=E+E_{1drop}+E_{2drop}-E_{drop-drop}-E_{pore-pore}.$$

The terms in Expression (50) have the following meaning: the left part of (50) is the potential energy of a homogeneous liquid, which takes into account all interactions; the right side of (50) contains a sum that includes contributions related to the “separation” from a homogeneous liquid of two droplets with a free interface and free interface of the pores. 

To derive the interaction energy of two nanodroplets $E_{drop-drop}$ and the interaction energy of two nanopores $E_{pore-pore}$ (48), we first consider the interaction energy of a volume element that contains $n_{0}d^{3}r_{1}$ atoms, with a droplet of radius a:

(51) $$E_{el-drop}\left(R_{1}\right)=n_{0}^{2}dV\int_{V}d^{3}r_{2}F_{2}\left(\left|R_{1}-r_{2}\right|\right)\Phi \left(\left|R_{1}-r_{2}\right|\right)$$

where V is a droplet volume; and $F_{2}\left(\left|R_{1}-r_{2}\right|\right)$ is a pair distribution function of atoms in a homogeneous liquid.

We assume that the droplet is centered in the reference coordinate system and the volume element is located along the OZ axis at distance $R_{1}>a.$ In a spherical coordinate system, Expression (51), similar to (5), takes the form:

(52) $$\begin{array}{c} E_{el-drop}\left(R_{1},a\right)=\frac{\pi n_{0}dV_{1}}{R_{1}}\Theta \left(R_{1}-a\right)\int_{R_{1}-a}^{R_{1}+a}dRR\Phi \left(R\right)F_{2}^{\left(0\right)}\left(R\right)\times \\ \times \left[a^{2}-R_{1}^{2}-R^{2}+2RR_{1}\right]. \end{array}$$

Let us assume that the center of the second droplet with radius b is located along OZ at a point with coordinate d Then, the interaction energy of these two droplets with center distances d>a+b, using (52), can be written as:

(53) $$E_{drop-drop}\left(a,b,d\right)=\int dV_{1}E_{el-drop}\left(R_{1},a\right).$$

Integrating over spherical variables (53) will take the form:

(54) $$\begin{array}{c} E_{drop-drop}\left(a,b,d\right)=\frac{\pi^{2}n_{0}^{2}}{2d}\int_{d-b}^{d+b}dR_{1}\left[b^{2}-{\left(d-R_{1}\right)}^{2}\right]\times \\ \int_{R_{1}-a}^{R_{1}+a}dRRF_{2}\left(R\right)\Phi \left(R\right)\left[a^{2}-{\left(R_{1}-R\right)}^{2}\right]. \end{array}$$

The Expression (54) allows calculating a component of the interaction force between two nanodroplets along the OZ direction

(55) $$F_{z}\left(a,b,d\right)=-\frac{\partial }{\partial d}E_{drop-drop}\left(a,b,d\right).$$

A continuous density distribution is assumed in the boundary case of a continuum model, and it is not accounted for in the discreet structure of the media. The expressions derived above for the interaction energy of two nanodroplets and two nanopores can be easily reduced to the continuum case by neglecting the correlations and assuming $F_{2}\left(R\right)=1$. Using the Lennard–Jones potential for the interaction energy of atoms, and integrating Expression (54) over R, we obtain:

(56) $$\begin{array}{c} E_{drop-drop}\left(a,b,d\right)=\frac{2\pi^{2}n_{0}^{2}\varepsilon \sigma^{6}}{d}\int_{d-b}^{d+b}dR_{1}\left[b^{2}-{\left(d-R_{1}\right)}^{2}\right]\times \\ \{-\left(a^{2}\sigma^{6}/10\right)\left({\left(R_{1}+a\right)}^{-10}-{\left(R_{1}-a\right)}^{-10}\right)+\left(a^{2}/4\right)\left({\left(R_{1}+a\right)}^{-4}-{\left(R_{1}-a\right)}^{-4}\right)+\\ +\left(R_{1}^{2}\sigma^{6}/10\right)\left({\left(R_{1}+a\right)}^{-10}-{\left(R_{1}-a\right)}^{-10}\right)-\left(2R_{1}\sigma^{6}/9\right)\left({\left(R_{1}+a\right)}^{-9}-{\left(R_{1}-a\right)}^{-9}\right)+\\ +\left(\sigma^{6}/8\right)\left({\left(R_{1}+a\right)}^{-8}-{\left(R_{1}-a\right)}^{-8}\right)-\left(R_{1}^{2}/4\right)\left({\left(R_{1}+a\right)}^{-4}-{\left(R_{1}-a\right)}^{-4}\right)-\\ -\left(1/2\right)\left({\left(R_{1}+a\right)}^{-2}-{\left(R_{1}-a\right)}^{-2}\right)+\left(2R_{1}/3\right)\left({\left(R_{1}+a\right)}^{-3}-{\left(R_{1}-a\right)}^{-3}\right)\}. \end{array}$$

Formula (56) can be simplified by integration over $R_{1}$

(57) $$\begin{array}{c} E_{drop-drop}\left(a,b,d\right)=\frac{2\pi^{2}n_{0}^{2}\varepsilon \sigma^{6}}{d}\left(b^{2}-d^{2}\right)\times \\ \left\{\frac{\sigma^{6}a}{40}f_{8}^{+}+\frac{11}{630}\sigma^{6}f_{7}^{-}-\frac{a^{2}}{12}f_{3}^{-}-\frac{a^{2}}{12}f_{3}^{+}+\frac{a}{3}f_{2}^{-}+\frac{a}{3}f_{2}^{+}+\frac{1}{12}f_{1}^{-}\right\}+\\ +4\pi^{2}n_{0}^{2}\varepsilon \sigma^{6}\{-\frac{\sigma^{6}a^{3}}{20}f_{9}^{-}+\frac{31}{360}a^{2}\sigma^{6}f_{8}^{-}+\frac{1}{90}\sigma^{6}a^{3}f_{9}^{+}-\frac{1}{360}a\sigma^{6}f_{7}^{+}-\\ -\frac{1}{2160}\sigma^{6}f_{6}^{-}-\frac{1}{24}a^{3}f_{3}^{+}-\frac{1}{12}a^{2}f_{2}^{-}+\frac{1}{12}af_{1}^{-}-\frac{1}{12}\ln\left|\frac{a\delta }{\gamma \beta }\right|\}-\\ -\frac{2\pi^{2}n_{0}^{2}\varepsilon \sigma^{6}}{d}\{-\frac{\sigma^{6}a^{3}}{360}f_{8}^{+}+\frac{\sigma^{6}a^{2}}{168}f_{7}^{-}-\frac{\sigma^{6}a}{360}f_{6}^{+}-\frac{41\sigma^{6}}{1800}f_{5}^{-}-\\ -\frac{a^{3}}{4}f_{2}^{+}+\frac{55a^{2}}{36}f_{1}^{-}\}. \end{array}$$

where we denoted:

(58) $$\begin{array}{c} f_{1}^{\pm }=\alpha^{-1}-\beta^{-1}\pm \gamma^{-1}\pm \delta^{-1},\;f_{2}^{\pm }=\alpha^{-2}-\beta^{-2}\pm \gamma^{-2}\pm \delta^{-2},\\ f_{3}^{\pm }=\alpha^{-3}-\beta^{-3}\pm \gamma^{-3}\pm \delta^{-3},\;f_{5}^{\pm }=\alpha^{-5}-\beta^{-5}\pm \gamma^{-5}\pm \delta^{-5},\\ f_{6}^{\pm }=\alpha^{-6}-\beta^{-6}\pm \gamma^{-6}\pm \delta^{-6},\;f_{7}^{\pm }=\alpha^{-7}-\beta^{-7}\pm \gamma^{-7}\pm \delta^{-7},\\ f_{8}^{\pm }=\alpha^{-8}-\beta^{-8}\pm \gamma^{-8}\pm \delta^{-8},\;f_{9}^{\pm }=\alpha^{-9}-\beta^{-9}\pm \gamma^{-9}\pm \delta^{-9},\;\\ \alpha =d+b+a,\;\beta =d-b+a,\;\gamma =d+b-a,\;\delta =d-b-a. \end{array}$$

The result in (57) corresponds to the continuum approximation for the interaction energy of two droplets of liquid with parameters $E_{drop-drop}\left(a,b,d\right)$. It accounts for attractive and repulsive parts of molecule interactions and neglects correlation effects.

The absorption problems in equilibrium liquid–vapor systems require knowledge of the nanodroplet interaction energy with the flat liquid interface. Using Expression (54) for the interaction energy of two droplets, we can investigate the boundary case of the droplet–semi-bounded liquid interaction.

These boundary case calculations of the droplet interaction energy with the semi-bounded liquid can be performed assuming that the distance between droplet centers d→∞ and droplet radius b→∞, and d−b=const. We denote distance d−b=D, which in our boundary case corresponds to the distance from the center of the droplet with radius a to the interface of semi-bounded liquid. The calculation of the limit $\underset{\begin{array}{l} d\to \infty ,b\to \infty \\ d-b=D \end{array}}{\lim}E_{drop-drop}\left(a,b,d\right)$ is not dependent on the internal integral in (54). Thus:

(59) $$\underset{\begin{array}{l} d\to \infty ,b\to \infty \\ d-b=D \end{array}}{\lim}\frac{1}{d}\left[b^{2}-{\left(d-R_{1}\right)}^{2}\right]=2R_{1}-2D.$$

Using the result in (58), the interaction energy of the droplet of radius a with a semi-bounded liquid at a distance D will take the form:

(60) $$\begin{array}{c} E_{drop-semi}\left(a,b,d\right)=\pi^{2}n_{0}^{2}\int_{D}^{\infty }dR_{1}\left(R_{1}-D\right)\times \\ \times \int_{R_{1}-a}^{R_{1}+a}dRRF_{2}\left(R\right)\Phi \left(R\right)\left[a^{2}-{\left(R_{1}-R\right)}^{2}\right]. \end{array}$$

The derived Expression (60) takes into account the paired interparticle interactions by means of potential Φ(R) and the paired interparticle correlations by means of $F_{2}\left(R\right)$. From (59), we can derive an explicit expression for the interaction energy of the nanodroplet with a semi-bounded liquid in continuum approximation.

**Figure 11:**
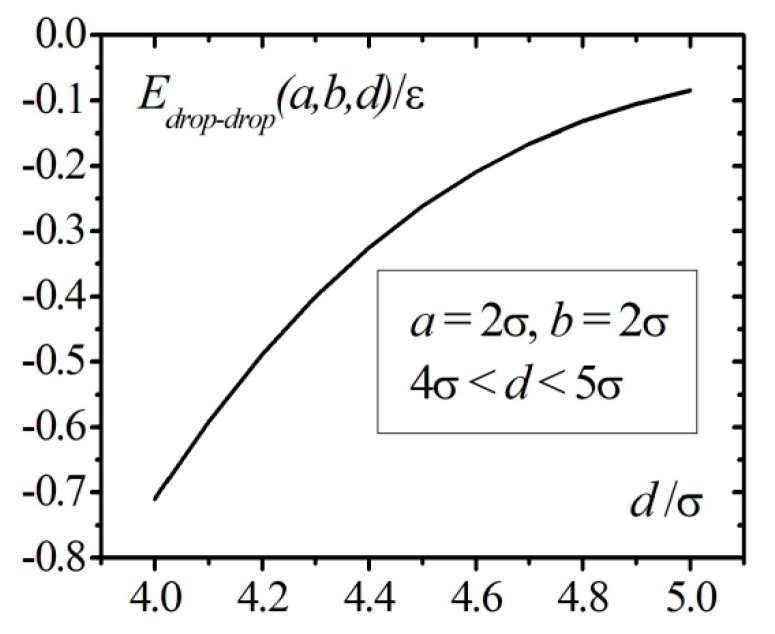
Dependence of the interaction energy of two ${}^{4}He$ nanodroplets with radii a=2σ.

Figure 11 shows the results of numerical calculations of the interaction energy of two ${}^{4}He$ nanodroplets with the same radii a=2σ as a function of the distance between the centers of the droplets 4σ<a<5σ in accordance with the Formula (54). The interaction energy of nanodroplets is negative and rapidly decreases with increasing distance between the centers of the droplets.

## 5. The Effective Hamiltonian of Aerosols with Liquid Nanodroplets

We use the derived results to build the effective Hamiltonians of nanodispersed two-phase liquid–gas systems. Our approach accounts for the effects of the paired interparticle interactions and correlations in calculations of the molecules’ interaction energies with droplets, pairs of droplets, and the surface energy of the droplets.

Let us assume the existence of an aerosol of nanodroplets of different sizes in the gas phase. For simplicity, we assume that this two-phase system consists of atoms (molecules) of the same kind and has temperature T. Based on the results derived in the previous sections, the effective Hamiltonian of this system $H_{eff}$, including accounting for the paired interparticle interactions and correlations, can be written as:

(61) $$\begin{array}{c} H_{eff}=\frac{3}{2}Nk_{B}T+K+\varepsilon_{l}\sum_{j=1}^{N_{d}}V_{j}+\varepsilon_{g}\left(V-\sum_{j=1}^{N_{d}}V_{j}\right)+\sum_{j=1}^{N_{d}}\sigma_{j}S_{j}+\\ +\;\sum_{i=1}^{N_{G}}\sum_{j=1}^{N_{d}}\;E_{a-d}\left(R_{ij},a_{j}\right)+\frac{1}{2}\;\sum_{k=1}^{N_{d}}\sum_{m=1}^{N_{d}}E_{drop-drop}\left(a_{k},b_{m},d_{km}\right) \end{array}$$

where $\frac{3}{2}Nk_{B}T$ is the kinetic energy of all molecules of aerosol; N is the total number of molecules in gas and droplets; K is the kinetic energy of the translational and rotational motion of all droplets; $\varepsilon_{l},\;\varepsilon_{g}$ are the bulk energy densities of droplets and gas (30); V is the volume of the system; $V_{j}$ is the volume of the j-th droplet; $N_{d}$ is the number of droplets; $N_{G}$ is the number of molecules of the gas phase; $\sigma_{j},\;S_{j}$ is the surface energy and the surface area of the jth droplet, respectively (27); $E_{a-d}\left(R_{ij},a_{j}\right)$ is the interaction energy between the i-th gas molecule and the j-th droplet (5), $a_{j}$ is the radius of the j-th droplet; $R_{ij}$ is the distance between the i-th gas molecule and the center of the j-th droplet; and $E_{drop-drop}\left(a_{k},b_{m},d_{km}\right)$ is the interaction energy of two droplets with radiuses $a_{k}$ and $b_{m}$, and a center distance $d_{km}>a_{k}+b_{m}$ (50).

The expression for the effective Hamiltonian of an aerosol (61) can be generalized for the case of multicomponent mixtures with atoms (molecules) of different kinds. Formula (61) is derived for the set of independent variables: temperature, number of atoms (molecules) in a system in total and in gas phase, size of droplets, and density of the number of atoms in liquid and gas. In real experiments with macroscopic aerosol systems, statistical datasets exist that describe the dispersion of droplet sizes and the possible disposition in external fields. Knowledge of these statistical datasets is necessary for the averaging of (61) and calculation of aerosol energy.

The prediction of aerosol behavior requires knowledge of droplets’ size evolution, their concentration, the collisions results with possible coagulation, and accounting for the condensation and evaporation effects on and from the droplets’ surface. The time evolution of the droplet’s size distribution is described by the generalized integral–differential dynamic equation, which takes into account the balance of the number of atoms (molecules) of gas and the number of droplets, which may vary due to condensation, evaporation, and the coagulation of droplets [25,26,27,28].

The condensation phenomena, in addition to evaporation from the droplet’s surface, play an essential role in many technological processes. An overview of the existing approaches to the description of the evaporation and condensation phenomena is provided in work [29]. The majority of research papers use the equations of macroscopic mechanics of continuum media and thermodynamics. However, the framework of these approaches does not allow the formulation and establishment of the boundary conditions in the droplet’s near-surface area, where the application of the macroscopic equations of thermodynamics, heat transfer, and diffusion is problematic [29]. The microscopic approach developed in the current paper allows, at the molecular level, accounting for the interaction energies of atoms (molecules) with a liquid droplet, and it takes into account paired interparticle interactions and correlations with an arbitrary atom (molecule) disposition relative to the droplet. The derived expressions for $E_{a-d}\left(R_{ij},a_{j}\right)$ allow research on the intermolecular forces that act on a separate particle at an arbitrary position. They also allow the development of the statistical theory of equilibrium evaporation and condensation processes for arbitrary temperatures and densities of liquid and gas in multicomponent systems with droplets of an arbitrary size. In the problems of the adhesion of liquid droplets with other molecular structures, the interaction energy $E_{a-d}\left(R_{ij},a_{j}\right)$ plays an essential role and serves as a basis for further calculations.

The atom (molecule) work function from the droplet can be described as:

(62) $$A_{a-d}=E_{a-d}\left(\infty ,a\right)-E_{a-d}\left(0,a\right).$$

Expression (62) can be used in adhesion problems of atoms (molecules) with droplets and thermodynamics of aerosols. The sign of the atom work function of a droplet is dependent on thermodynamic conditions and may be negative. At condition $A_{a-d}>0$, condensation processes mostly take place, whereas at thermodynamic conditions when $A_{a-d}<0$, the evaporation effects are active. The case $A_{a-d}=0$ corresponds to the dynamic equilibrium of the evaporation and condensation processes. 

In molecular biology problems, the adhesion of large molecules (viruses) to the surface of cells and the adhesion of cells to each other play an essential role [30]. Current progress in the physics, chemistry, and experimental techniques with nanodroplets, cells, and viruses allows measurement of the adhesion forces of nanodroplets and viruses (virions) [30,31]. However, contemporary research on the energy and adhesion forces of nano-objects consists of only phenomenological developments. In this phenomenological state, the development of the molecular structures’ adhesion problems and nanodroplets of liquid, or the flat liquid interface, are important for the spread of viruses in aerosols. Within the framework of the approach developed in the current article, we can write the expression for the interaction energy of the multi-atom molecule, consisting of $N_{str}$ atoms, with a liquid droplet of radius $a_{j}$, as follows:

(63) $$E_{drop-str}=\sum_{i=1}^{N_{str}}E_{a-d}\left(R_{ij},a_{j}\right),$$

where $R_{ij}$ is the distance between the i-th atom of the structure and the j-th droplet.

Discussion about the adhesion mechanism of cells and viruses is long standing. The main question is whether the adhesion occurs at the direct contact of two objects or by means of intermediate molecular structures [30,31]. The same question concerns the mechanism of adhesion of two nanodroplets. The adhesion energy of two droplets with radiuses $a_{1},\;a_{2\;}$, which are in contact with each other, corresponds to the interaction energy $E_{drop-drop}\left(a_{1},a_{2},d\right)$, where d is the center distance between droplets.

The energy of indirect interaction of two nanodroplets with radiuses $a_{1},\;a_{2\;}$ over a molecular structure with $N_{str}$ atoms can be written:

(64) $$E_{drop-drop}^{ind}\left(a_{1},a_{2},d\right)=\sum_{k=1}^{2}\sum_{i=1}^{N_{str}}E_{a-d}\left(R_{il},a_{j}\right).$$

Formula (64) for the energy of the indirect interaction corresponds to the adhesion energy for the system of three bodies, and it can be defined as a separation work for all three components of the system to infinite distances. The boundary cases of Formula (64) describe the energy of indirect interaction of two half-spaces over the molecular structure between them. It is also important to note that all derived interaction energy Expressions (61)–(64) explicitly account for all paired interparticle interactions and correlations.

The collisions of droplets play an important role in the aerosol coagulation phenomena. The interaction energy of two droplets contains the direct interaction component $E_{drop-drop}\left(a_{1},a_{2},d\right)$ and the component of indirect interaction $E_{drop-drop}^{ind}\left(a_{1},a_{2},d\right)$ with the surroundings (atoms, molecules, molecular structures). For nanodroplet coagulation problems, one has to take into account both components. For the problems of cell and virus adhesion calculations, the comparison of direct and indirect interactions is required.

## 6. Discussion

The widely used continuum model of condensed systems has a limited application in the description of atoms’ interactions with condensed bodies [11,12]. The model neglects the discrete atomic structure of condensed bodies, interatomic correlations, and the ability of atoms to penetrate the condensed bodies. This is clearly visible with an example of interaction of separate atoms with droplets of liquids (Figure 6). The neglect of interatomic correlations leads to an unsatisfactory description of the physics of processes that are responsible for the dynamic equilibrium of the liquid–gas system.

When calculating the surface properties of condensed systems that are different in nature, the Fowler approximation is essential, because it correctly takes into account the basic surface contributions to different thermodynamic quantities. Corrections to this approximation, due to the difference between the real density profile in the near-surface layer and the stepped layer, are of an additive nature when calculating thermodynamic functions. Going beyond the Fowler model in our problem will not change the main results and conclusions of the work. Slight changes in the energy dependence of the interaction between the atom and the nanodroplet can be expected in the near-surface region.

For condensed systems, a characteristic property is the same order of magnitude of the average kinetic and potential energies of atoms and molecules. For nano-objects, due to the large proportion of particles present in the near-surface layer, interatomic correlations can be of great importance for the formation of a self-consistent potential, which ensures the stability of the object in relation to the decay of constituent atoms or molecules. The processes of nucleation, and the mechanisms of growth and evolution of nanostructures under conditions of microscopic instability, are an integral part of the physics of phase transitions of the first kind. From the point of view of kinetics, the processes of nucleation of nano-objects take place with the participation of each individual atom, which evolves in the field of other atoms. The evolution of a single atom depends on the self-consistent field formed by other atoms and correlations with neighboring atoms. Stability of processes and their direction are also important for the growth or decay of nano-objects. The microscopic nature of the mono-atomic mechanisms of growth or decay of nanostructures, when the curvature of the surface of the new nanophase is significant, has been insufficiently studied. The solution of kinetic problems of this type is possible by means of theoretical calculations of the atom’s interaction energy with the inhomogeneous environment at a nanometer scale. A proper description of the multiscale processes of nanostructure growth also requires knowledge of the energy balance of the entire nanostructure, which requires energy calculations of highly heterogeneous nanoscale systems.

The distribution functions method of groups of atoms allows the equilibrium properties of the liquid–gas interface to be expressed in terms of potentials of interatomic interactions and distribution functions of atoms. Among the most important thermodynamic functions are the surface energy and surface tension of flat and curved surfaces. When calculating the surface properties of the liquid–gas interface, the use of the Fowler approximation [18,19] for unary and binary atom distribution functions allows us to make estimates of surface contributions with considerable accuracy [10,16,19,22]. Corrections to the Fowler approximation strongly depend on the approximation methods for the non-central part of the pair distribution function and on the choice of the atomic density profile [10,16,19,22]. The main efforts in modern statistical physics relating to two-phase liquid–gas systems are aimed at calculating the atom’s density profile near the phase interface and calculating its surface energy and surface tension.

In the current work, within the framework of the distribution function method of the group of particles and Fowler’s approximation, we derive the expressions for the atom interaction energy with a nanodroplet of simple liquid. The derived formulation is applied for the calculations of the nanodroplets of liquid helium. The developed approach has similarities with the functional density method (FDM) [32,33,34,35,36]; however, it additionally takes into account the energies of the paired interparticle correlations, which are problematic in FDM. The analysis of different approaches for the accounting of pair correlation energies in the FDM in application to liquid helium is described in paper [36]. Assembling the expression for the energy of pair correlations only by means of the atom’s paired interaction energy and local densities of the number of atoms requires the introduction of the non-physical “screening” concept of Lennard–Jones potential at small distances, to avoid the divergence problem of the corresponding integrals [36]. In fact, it is necessary to introduce additional parameters that cannot be determined within the density functional method itself. As a result, extra designations for Lennard–Jones potential are introduced in different versions of FDM at short distances in an attempt to converge the integrals corresponding to the indirect accounting for the short-range paired interatomic correlations. The distribution function method used above for groups of particles avoids the necessity of expanding the studied quantities into gradient series.

The model calculations performed above for the size dependence of the atoms’ interaction with a nanodroplet of helium indicates a significant influence of the saturation effect of the single atomic energy inside the droplet. The short range of interatomic interactions results in the rapid achievement of the asymptotic value of the atoms’ energy at the center of the droplet during the growth of the droplet size. The formation of the bulk properties of helium nanodroplets occurs at radii in the order of 4σ and the number atoms of around 101. It should be noted that the pair distribution function of atoms in liquids at distances 4σ is almost equal to one, which corresponds to the absence of correlations in the spatial positions of the pair of atoms. Therefore, it is possible to reach conclusions about the relationship between the radii of liquid nanodroplets, at which the volumetric properties of the liquid are formed, and the characteristic distances at which the paired interatomic correlation in liquids is lost. This conclusion also easy to see in Figure 5, from which it follows that most atoms in droplets of the specified size have the same one-atomic potential as in a homogeneous liquid. The saturation effect is also observed for the thickness of the near-surface region, in which the monoatomic potential changes its value from its “bulk” value to zero outside the droplet. The depth of the potential well, in which the atom moves inside the helium droplet, for the droplets with radii of the order a>4σ, has a magnitude 5ε≈50K, which significantly exceeds the thermal energy of the atoms (T=2.2K).

The kinetic energy of helium atoms contains the classical thermal contribution and the quantum contribution. Calculations of the kinetic energy of helium atoms in a wide range of densities and temperatures using the Monte Carlo quantum method [35,36] indicate the importance of accounting for the quantum contributions. At the selected temperature and density of liquid helium, the kinetic energy, according to the results in [36], is of the order 15K, which means that the main contribution to the kinetic energy is made by quantum effects. A comparison of the kinetic energy with the depth of the potential well shows that the atoms are localized in the droplet and that helium nanodroplets tend to be resistant to spontaneous decay due to thermal fluctuations and the emission of individual atoms.

## 7. Conclusions

In this paper, in the framework of the correlation theory of inhomogeneous liquids, general expressions are obtained for the volume and surface contributions to the energy of a droplet as a function of its radius. In Fowler’s approximation, all contributions are expressed in the form of one-time integrals, which significantly simplifies the model calculations of the dimensional dependence. The dimensional dependence of the surface energy of spherical droplets of simple dielectric liquids as a function of the radius in the Fowler approximation is calculated. In the extreme case of large values of the radius of a droplet, its surface energy approaches its value for a flat surface. The strong dimensional dependence of the surface energy of the droplet is observed in the region of nanometer droplet size. The developed approach is applied to the calculation of the surface energy of a two-phase system of liquid nanodroplet–gas phase, taking into account paired interparticle interactions and correlations. The dimensional dependence of the surface energy of a nanodroplet in a gas is similar to the dimensional dependence of a nanodroplet in a vacuum.

This paper shows the possibility of taking into account paired interparticle interactions and correlation effects when calculating the interaction energy of pairs of droplets of arbitrary size. The energy of the interaction of two droplets in the Fowler approximation is written as a double integral. An explicit expression for the interaction energy of two droplets in the continuum approximation is obtained, which allows us to investigate the importance of taking into account the effects of paired intermolecular correlations in comparison with continuum models. As a boundary case, the expression for the energy of interaction of a droplet and a semi-bounded liquid is obtained, taking into account the effects of paired interparticle correlations.

On the basis of the constructed theory, the properties of nanometer air dispersal systems with arbitrary droplet dispersion and for arbitrary multicomponent mixtures of liquids can be calculated. By means of the effective Hamiltonian of the aerosol of liquid droplets (61), it is possible to investigate in more detail the kinetic problems of evaporation and condensation on the surfaces of droplets and the lifetime of nanodroplets during their evaporation.

Within the framework of the developed approach, it is possible to study the interaction of liquid nanodroplets with foreign molecular structures (e.g., virions), coagulation of molecular structure and nanodroplets, and evaporation and condensation processes on droplets, provided that the molecular structure is present inside the droplet. These issues are all currently unresolved. The important theoretically unresolved issues are the evolution processes of evaporation and condensation on a nanodroplet that is in contact with different media (wood, plastic, metal), and the lifetime of the droplet with a molecular structure inside different surfaces. The answers to these questions are important in the problems related to the spread of viruses.

## Data Availability

Data available in a publicly accessible repository.
